# Artificial Intelligence in Colonoscopy: A Systematic Review of Adenoma Versus Polyp Detection Rates

**DOI:** 10.7759/cureus.98528

**Published:** 2025-12-05

**Authors:** Waseem Rabba, Fatima Asif, Muhammad Y Younis, Haris Nasrullah, Laraib Fatima, Muhammad A Arif

**Affiliations:** 1 Health Sciences, McMaster University, Vaughan, CAN; 2 Internal Medicine, Shrewsbury and Telford NHS Trust, Telford, GBR; 3 Gastroenterology, Sahara Medical College, Narowal, PAK; 4 Internal Medicine, Barnsley Hospital, Barnsley, GBR; 5 Medicine and Surgery, Nawaz Sharif Medical College Gujrat, University of Health Sciences Lahore, Gujrat, PAK; 6 Internal Medicine, Royal Blackburn Hospital, Blackburn, GBR

**Keywords:** adenoma detection rate, artificial intelligence, colonoscopy, computer-aided detection, polyp detection rate

## Abstract

Colonoscopy is the gold standard in the prevention of colorectal cancer, but the miss rates of adenoma are high, which restricts its efficacy. To improve lesion recognition, artificial intelligence (AI), especially computer-aided detection (CADe) systems, has been introduced. The aim of this systematic review was to compare AI-assisted colonoscopy in terms of its ability to improve adenoma detection rate (ADR) and polyp detection rate (PDR). An extensive search was performed on PubMed, Embase, and Cochrane Library from 2015 to 2025. There were 17 randomized controlled trials (RCTs) comparing the use of AI-assisted colonoscopy with normal colonoscopy. The methodological quality measure of the included RCTs was Cochrane Risk of Bias 2.0 (RoB 2.0), which subdivided the studies based on low risk, some concerns, or high risk of bias based on whether they were biased in this or that domain. The robVis tool was used to produce the visual summaries. AI-aided colonoscopy effectively enhanced both adenoma detection rate (ADR) and polyp detection rate (PDR) in all of the included studies over conventional colonoscopy. In adenoma detection, accuracy was more than 85%, and in polyp detection, more than 90%. The advantage was also found especially in the detection of small and flat adenomas, which are very often missed in routine practice. The use of AI in colonoscopy is strongly associated with an increase in the detection rate of adenoma and polyps, minimizing the risk of underdiagnosis. The results highlight the clinical promise of AI in the form of a decision-support tool across gastroenterologists and suggest that AI can be applied to enhance the outcomes of preventive and screening colorectal cancer. Future research should be cost-efficient and practical, and combined with some clinical activities.

## Introduction and background

Colorectal cancer (CRC) is a type of malignancy that is prevalent across the country and is also a major cause of cancer-associated deaths [1]. The first and most important approach to preventing the development of CRC is early detection and ablation of adenomas and polyps by means of colonoscopy [2]. Whereas traditional colonoscopy is the gold standard of colonoscopy screening, it suffers from the human factor of exhaustion, lack of experience, or poor technique, leading to inadvertent lesion misses and hence non-uniform detection rates across operators. Research has shown that traditional colonoscopy is unable to detect up to 25% of adenomas, especially small, flat, or sessile lesions [3, 4]. This diversity in adenoma detection rates (ADR) and polyp detection rates (PDR) carries important clinical consequences, since unidentified lesions may advance to higher-grade neoplasia or to CRC. Thus, an increased demand is emerging for tools that can help to standardize detection and improve the accuracy of endoscopic examinations [5, 6].

Deep learning algorithms and artificial intelligence (AI) have become potentially valuable supplements to gastrointestinal endoscopy [7]. Computer-aided detection (CADe) is an AI-based system that can aid endoscopists during colonoscopy by identifying any potential lesions. They are able to analyze high-definition video images with great sensitivity and hence detect subtle adenomas and polyps that otherwise would have been ignored [8, 9].

AI-assisted colonoscopy is a highly effective technique to enhance ADR and PDR over traditional techniques [10]. Indicatively, randomized trials have found ADR improvements of about 19% to almost 30% and PDR improvements of 30 to above 45 [11, 12]. AI support has specifically been useful in the identification of small or flat lesions, which the human endoscopist finds harder to identify in traditional ways [13].

AI-driven colonoscopy can reduce colorectal cancer through more accurate detection and ablation of precancerous lesions, improve patient outcomes, and harmonize the quality of screening among care settings. The systematic review aims to compare and contrast the effects of the use of AI-assisted colonoscopy on the detection rates of adenoma and polyps. This review aims to present a summary evaluation of the role of AI in improving the performance of colonoscopes, its clinical advantages and disadvantages, and future opportunities to develop research and practice in the field.

## Review

Methodology

The review adhered to Preferred Reporting Items for Systematic Reviews and Meta-Analyses (PRISMA) guidelines and used the PICO framework to formulate the research question as described in Table 1 [14, 15].

**Table 1:** PICO framework MeSH - Medical Subject Headings; CADe - computer-aided detection

Concepts	Text words	Controlled vocabulary (MeSH terms)
Population/ problem	“Colonoscopy” OR “Colorectal cancer screening” OR “Adenoma” OR “Polyp”	"Colonoscopy" [MeSH] OR "Colorectal Neoplasms" [MeSH] OR "Adenoma" [MeSH] OR "Polyps" [MeSH]
Intervention	“Artificial Intelligence” OR “Computer-Aided Detection” OR “Deep Learning” OR “CADe”	"Artificial Intelligence" [MeSH] OR "Machine Learning" [MeSH] OR "Computer-Assisted Diagnosis" [MeSH]
Comparison	No Intervention	No Intervention
Outcome	“Adenoma Detection Rate” OR “Polyp Detection Rate” OR “Missed Lesions” OR “Diagnostic Accuracy”	"Sensitivity and Specificity" [MeSH] OR "Diagnosis, Computer-Assisted" [MeSH] OR "Early Detection of Cancer" [MeSH]

Objective

The objective of the study was to evaluate the effectiveness of artificial intelligence-assisted colonoscopy in improving the adenoma detection rate (ADR) and polyp detection rate (PDR).

Search Strategy and Search String

Electronic databases searched included PubMed, Embase, and Cochrane. Searches were systematically performed using Medical Subject Headings (MeSH) terms. Boolean operators "AND" and "OR" were applied to appropriately link search terms and ensure broad and precise coverage of the literature. ("Colonoscopy" (MeSH) OR "Colorectal Neoplasms" (MeSH) OR adenoma OR polyp) AND ("Artificial Intelligence" (MeSH) OR "Computer-Assisted Diagnosis" (MeSH) OR "Deep Learning" OR "CADe") AND ("Adenoma Detection Rate" OR "Polyp Detection Rate" OR "Missed lesions" OR "Diagnostic Accuracy").

Study Selection Criteria

The studies had to assess the use of artificial intelligence (AI) in colonoscopy for adenoma detection rate (ADR) and/or a higher polyp detection rate (PDR). Only the randomized controlled trials in the English language were included so that the methodological rigor would be assured and so that the results could be compared. Studies that were not interested in AI-based detection, including those that evaluated only computer-aided diagnosis (CADx) to determine histological characterization, non-endoscopic imaging modalities, or only experimental algorithms with no clinical implementation, were excluded. Abstracts of conferences, editorials, case reports, and narrative reviews were also not included because they do not provide enough methodological information or outcome measures. In order to increase the credibility of the synthesis, the articles discussing pediatric populations, non-human models, or interventions not related to colonoscopy were eliminated. The articles that only described technical performance with no reports on ADR or PDR results were also eliminated.

Study Selection Process

The studies were selected in two ways. Two independent reviewers screened the titles and the abstracts of all the records obtained in the first phase to identify the potential eligibility. The second stage would involve the study of full texts of the identified articles with regard to set inclusion and exclusion criteria. The reviewers disagreed but were able to resolve the conflict through a discussion, and a third reviewer could be involved in this situation. Only articles that satisfied the inclusion criteria were chosen to proceed to the final synthesis [16].

Methodological Quality Assessment

The methodological quality measure of the included RCTs was Cochrane Risk of Bias 2.0 (RoB 2.0), which categorized the studies based on low risk, some concerns, or high risk of bias because they were biased regarding one or more domains [17]. Visual summaries were generated using the robvis tool [18].

Data Extraction and Synthesis

The data extract form was created to systematize in a standardized manner the following variables in each study: author/year, study design, sample size, baseline patient demographics, primary outcomes, secondary outcomes, and key findings. To identify the general patterns, an inductive, data-driven approach and the development of a comparative understanding of and between trials were used [19].

Ethical Consideration

**Figure 1:**
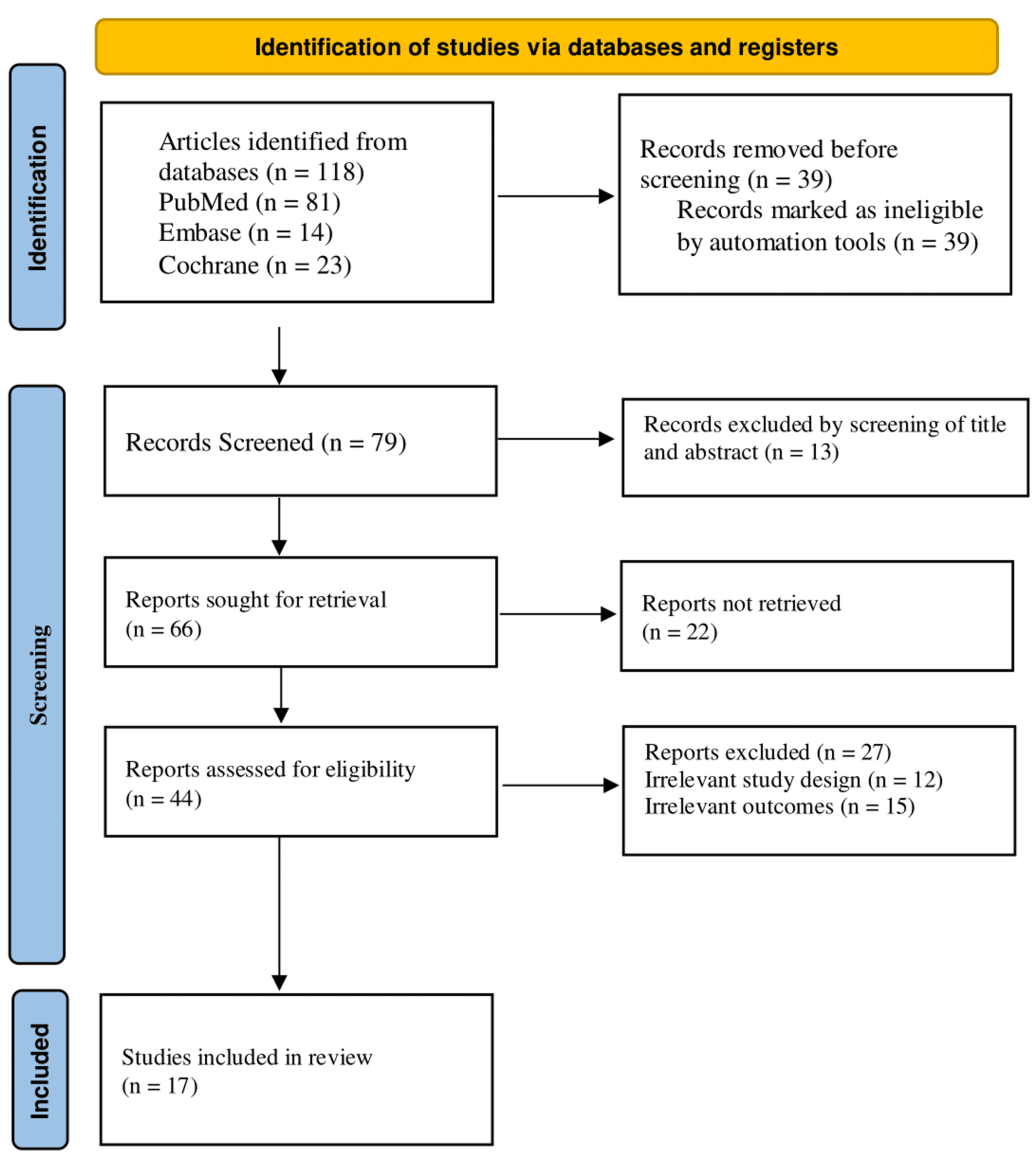
PRISMA flowchart PRISMA - Preferred Reporting Items for Systematic Reviews and Meta-Analyses

The review conformed to the Declaration of Helsinki, conformed to PRISMA recommendations, and was transparent, reproducible, and sound. It encouraged the use of evidence during the clinical decision-making process, and the results were reported in a peer-reviewed journal. Figure 1 shows the PRISMA flow diagram for the study selection process.

Results

A total of 118 articles were initially identified through database searches, including 81 from PubMed, 14 from Embase, and 23 from the Cochrane Library. Before screening began, 39 records were removed because they were marked as ineligible by automation tools. This left 79 records to be screened based on titles and abstracts. Following this screening process, 13 records were excluded. The remaining 66 reports were sought for retrieval. However, 22 of these could not be retrieved, leaving 44 reports to be assessed for eligibility. Of these, 27 reports were excluded due to irrelevant study design (n=12) or irrelevant outcomes (n=15). Ultimately, 17 studies met all inclusion criteria and were included in the final review.

Cochrane Risk of Bias Assessment

**Figure 2:**
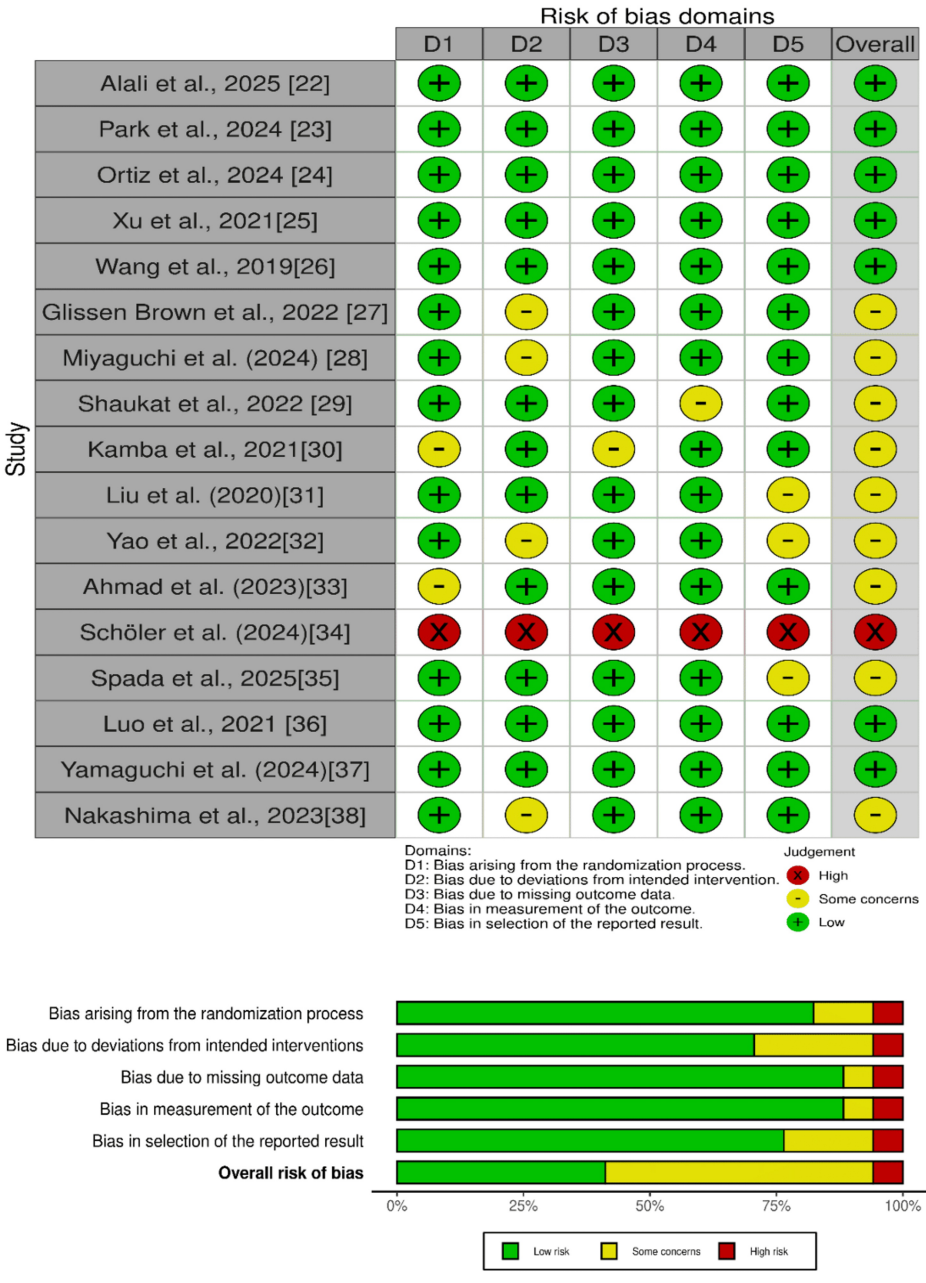
Cochrane Risk of Bias 2.0

Figure 2 shows the Cochrane systematic review assessing the risk of bias in studies, indicating high, low, or some concern based on selection, performance, detection, and reporting methods.

Based on the Cochrane RoB 2.0 assessment, seven studies had a low risk of bias, nine studies had a moderate risk of bias, and one study had a high risk of bias. Table 2 includes a summary of findings included in the review.

**Table 2:** Summary of findings included in review ADR - adenoma detection rate; PDR - polyp detection rate; APC - adenoma per colonoscopy; PPC - polyp per colonoscopy; HD-WL - high-definition white-light; CADe - computer-aided detection; WLE - white light endoscopy; AMR - adenoma miss rate; SSL - sessile serrated lesion; SSLDR - sessile serrated lesion detection rate; SSLPC - sessile serrated lesions per colonoscopy; CAQ - computer-aided quality improvement; ITT - intention-to-treat; BCSP - Bowel Cancer Screening Program; AI-C - colonoscopy with the assistance of artificial intelligence; CC - conventional colonoscopy; CUSUM - cumulative sum

Author & year	Study design	Sample size (n) / population characteristics	Type of colonoscopy (screening / surveillance)	AI system used	Primary outcome	Secondary outcomes	Key findings	Conclusion
Alali et al., 2025 [20]	Randomized-controlled trial (RCT)	102 patients; mean age 52.8 years, 50% male; average-risk, asymptomatic	Screening (94.1%), surveillance (5.9%)	CAD EYE (Fujifilm)	Adenoma detection rate (ADR)	Polyp detection rate (PDR), adenoma per colonoscopy (APC), polyp per colonoscopy (PPC), accuracy of polyp characterization	ADR: 47.1% (CADe) vs. 37.3% (HD-WL), p=0.32, RR=1.26 (0.80–2.00); PDR: 78.4% vs. 56.8%, p=0.02; PPC: 1.35 vs. 0.96, p=0.04; APC: 0.75 vs. 0.51, p=0.09; characterization accuracy: 79.7% vs. 76.9%, p=0.71	CADe showed a non-significant trend toward improved ADR but significantly increased PDR and PPC. AI did not improve polyp characterization accuracy. Larger studies are needed to confirm the effect on ADR in the Kuwaiti population.
Park et al., 2024 [21]	Prospective multicenter randomized controlled trial	805 patients; mean age 57.8 years; 386 males, 419 females; from 6 institutions	Screening (45.1-46.8%), symptom (30.0-37.4%), polyp surveillance (16.4-21.7%)	RetinaNet-based AI program (trained on still images and video)	Adenoma detection rate (ADR)	Polyp detection rate (PDR), biopsy rate, polyp location, size, and histology	ADR: 0.35 (AI) vs. 0.28 (control), p=0.03; PDR: 0.62 vs. 0.52, p=0.01; OR for PDR: 1.48 (95% CI: 1.11–1.99); OR for ADR: 1.40 (95% CI: 1.04–1.90); significant improvement in detection of polyps ≤0.5 cm (OR=1.53, p<0.01) and in sigmoid colon (OR=1.62, p=0.01)	AI-assisted colonoscopy significantly improved PDR and ADR, especially for small polyps (≤5 mm) and in the sigmoid colon, without increasing withdrawal time. AI is clinically beneficial for enhancing polyp detection.
Ortiz et al., 2024 [22]	Multicenter randomized controlled trial	430 patients; mean age 48.9 years; 59.5% female; Lynch syndrome carriers with pathogenic variants in MLH1, MSH2, MSH6, or EPCAM	Surveillance	GI Genius (Medtronic)	Mean number of adenomas per colonoscopy (APC)	ADR, serrated lesion detection, withdrawal time, false positive rate, optical diagnosis accuracy	APC: 0.64 (CADe) vs. 0.64 (WLE), adjusted RR=1.03 (95% CI 0.72–1.47), p=0.87; ADR: 32.7% vs. 36.1%, RR=0.92 (95% CI 0.75–1.13), p=0.42; higher histopathological false positives in CADe group: RR=2.93 (95% CI 1.48–5.79), p=0.002; increased detection of serrated lesions 5-9mm in CADe group: RR=2.31 (95% CI 1.15–4.63), p=0.02	CADe did not improve adenoma detection in Lynch syndrome patients compared to high-definition white light endoscopy. High-quality colonoscopy procedures remain essential for effective surveillance in this population.
Xu et al., 2021 [23]	Prospective multicenter randomized controlled trial	2352 patients; mean age ~51 years; gender-balanced; from 6 centers in China	Screening, surveillance, diagnostic	Custom RetinaNet-based AI system	Polyp detection rate (PDR)	Polyps per positive patient (PPP), Polyps per colonoscopy (PPC), Non-first polyps per colonoscopy (PPC-Plus)	PDR: 38.8% (AI) vs. 36.2% (control), p=0.183; PPC-Plus: 0.5 vs. 0.4, p<0.05; increased detection of diminutive polyps (76.0% vs. 68.8%, p<0.01) and flat polyps (5.9% vs. 3.3%, p<0.05); AI effect varied by center and was more pronounced with male endoscopists, shorter insertion time, longer withdrawal time, and older patients with larger waist circumference (OR=1.248, p=0.049)	AI assistance did not significantly improve overall PDR but increased detection of easily missed polyps (non-first, diminutive, and flat polyps). The effect of AI was influenced by endoscopist and procedure factors.
Wang et al., 2019 [24]	Prospective randomized controlled trial	1058 patients; mean age ~50-51 years; from a single center in China	Diagnostic (91.8-92.3% symptomatic), screening (7.7-8.2%)	Custom deep learning-based CADe system (Shanghai Wision AI)	Adenoma detection cate (ADR)	Polyp detection rate (PDR), mean number of polyps/adenomas per colonoscopy, false positive rate	ADR: 29.1% (AI) vs. 20.3% (control), p<0.001, OR=1.61 (95% CI 1.21–2.14); PDR: 45.0% vs. 29.1%, p<0.001, OR=1.995 (95% CI 1.53–2.54); mean adenomas per colonoscopy: 0.53 vs. 0.31, p<0.001; increase driven by diminutive adenomas (185 vs. 102, p<0.001); false positive rate: 0.075 per colonoscopy	The AI system significantly increased ADR and PDR, primarily by improving detection of diminutive adenomas. The system shows potential for clinical application in low ADR settings.
Glissen Brown et al., 2022 [25]	Prospective, multicenter, single-blind, randomized tandem colonoscopy trial	223 patients (after exclusions); 45.3% female, 67.7% Caucasian, 21% African American; mean age ~61 years	Screening (59.6%) and surveillance (40.4%)	EndoScreener (Shanghai Wision AI)	Adenoma miss rate (AMR)	SSL miss rate, APC, ADR, PDR, false positives/negatives	AMR: CADe-first 20.12% vs HD-WL-first 31.25% (OR 1.80; 95% CI 1.08–3.02; p=0.0247, p=0.0247). SSL miss rate: 7.14% vs 42.11% p=0.0482, p=0.0482). First-pass APC: 1.19 vs 0.90 (p=0.0323, p=0.0323). No significant difference in ADR.	CADe significantly reduces adenoma and sessile serrated lesion miss rates and increases adenomas per colonoscopy compared to HD-WL alone.
Miyaguchi et al. 2024 [26]	Prospective randomized controlled trial	800 patients (400 LCA, 400 LCI); mean age ~65–66 years; mixed indications (FIT+, symptoms, surveillance, screening)	Mixed (screening, surveillance, diagnostic)	CADEYE (EW10-EC02) by Fujifilm	Adenoma detection rate (ADR)	APC, cecal intubation time, withdrawal time, cleanliness, adenoma size/location/histology, SSL detection	ADR: LCA 58.8% vs LCI 43.5% (RR 1.351, 95% CI 1.176–1.551; p<0.001); APC: LCA 1.31 vs LCI 0.94 (p<0.001); SSL detection: LCA 4.0% vs LCI 1.0% (p=0.007); more small adenomas (≤5 mm, 6-9 mm) detected in LCA (p<0.001 and P = 0.04)	LCA significantly superior to LCI alone in ADR and APC, especially for small and sessile serrated lesions.
Shaukat et al., 2022 [27]	Prospective, multicenter, randomized controlled trial	1359 (mITT); aged ≥40, screening or surveillance (≥3 years), average-risk US population; 22 high-ADR endoscopists	Screening and Surveillance	SKOUT™ CADe (Iterative Scopes)	APC: 0.83 vs 1.05 (p=0.002); THR: 71.7% vs 67.4% (noninferiority p<0.001)	ADR, SSLDR, SSLPC, APP, WT, polyp detection rate, polyps per colonoscopy, surveillance interval	APC increased by 27% (0.22 absolute increase); ADR: 43.9% vs 47.8% (p=0.065); proximal polyp detection increased (p=0.001); no change in WT or procedure time	CADe significantly improves adenoma detection (APC) without reducing THR, supporting its use in routine clinical practice to potentially reduce interval colorectal cancers.
Kamba et al., 2021 [28]	Multicenter randomized controlled trial (tandem colonoscopy)	358 enrolled, 345 analyzed (172 CADe-first, 173 SC-first); mean age ~61 years; 76% male; 4 sites in Japan	Screening and surveillance	LPIXEL CADe system (based on YOLOv3)	Adenoma miss rate (AMR)	PMR, SSL miss rate, ADR, PDR, MAP, withdrawal time, surveillance interval change	AMR: CADe-first 13.8% vs SC-first 36.7% (p<0.0001, p<0.0001). SSL miss rate: 13.0% vs 38.5% (p=0.032, p=0.032). ADR: 64.5% vs 53.6% (p=0.036, p=0.036). PMR: 14.2% vs 40.6% (p<0.0001, p<0.0001).	CADe significantly reduces adenoma, polyp, and sessile serrated lesion miss rates, and increases adenoma detection rate in a diverse endoscopic setting.
Liu et al. 2020 [29]	Prospective randomized controlled trial	1026 patients (508 CADe, 518 control); mean age ~50–51 years; mixed indications (symptoms, screening)	Mixed (screening, diagnostic)	CADe system by Henan Xuanweitang Medical Information Technology Co., Ltd.	Adenoma detection rate (ADR)	Polyp detection rate (PDR), mean number of polyps/adenomas per colonoscopy, false-positive/false-negative rates	ADR: CADe 39.1% vs control 23.9% (OR = 1.64, 95% CI 1.201–2.220; p<0.001); Mean adenomas: CADe 0.52 vs control 0.34 (p<0.001); PDR: CADe 43.7% vs CON 27.8% (OR = 1.57, 95% CI 1.586–2.483; p<0.001); Mean polyps: CADe 0.87 vs control 0.57 (p<0.001); No difference in large adenomas (≥10 mm) (p>0.05)	The CADe system significantly increased the detection of polyps and adenomas, particularly small ones, and is feasible for use in colonoscopy.
Yao et al., 2022 [30]	Single-center, randomized, four-group parallel controlled trial	1076; adults ≥18, screening (89.13%), surveillance (9.48%), diagnostic (1.39%); Chinese population	Screening, surveillance, diagnostic	CADe (EndoAngel), CAQ (withdrawal speed monitoring)	ADR: Control 14.76%, CADe 21.27%, CAQ 24.54%, COMBO 30.60%	PDR, adenoma size/location, advanced ADR, SSL detection, withdrawal time, over-speed frames	COMBO ADR significantly higher than CADe (30.60% vs 21.27%, p=0.024, OR 1.284); no significant difference between COMBO and CAQ (p=0.213); CAQ reduced over-speed frames (P<0.001)	Combining CADe with CAQ significantly improves ADR without prolonging withdrawal time. CAQ enhances CADe efficacy, supporting combined use in clinical practice.
Ahmad et al. 2023 [31]	Prospective randomized controlled trial	614 patients (ITT), 579 (per-protocol); aged 60–74 (or 55 with specific criteria), NHS BCSP participants	Screening and surveillance	GI Genius (Medtronic)	Polyp detection rate (PDR)	ADR, SSL detection, significant polyp detection, polyps per colonoscopy, APC, SP6, procedure times	ITT: PDR 85.7% vs 79.7% (p=0.05); ADR 71.4% vs 65.0% (p=0.09); significant polyp detection 79.2% vs 71.6% (p=0.03). No difference in per-protocol analysis or procedure times.	CADe showed a borderline increase in PDR but no significant improvement in ADR. Limited benefit in a high-performing BCSP setting with frequent Endocuff Vision use.
Schöler et al. 2024 [32]	Prospective randomized controlled trial	240 patients (122 AI-C, 118 CC); mean age 66.4 years; mixed indications (alarm symptoms 56%, other 40%, screening 1%)	Mixed (diagnostic, surveillance, limited screening)	Fujifilm CAD EYE & Medtronic GI Genius	Adenoma detection rate (ADR)	PDR, APC, PPC, SSLDR, detection of small polyps (<5 mm), right-sided polyps, endoscopist experience subgroups	ADR: AI-C 43% vs CC 41% (RR 1.07, 95% CI 0.79–1.44; p=0.696); SSLDR: AI-C 22% vs CC 11% (RR 2.0, 95% CI 1.09–3.70; p=0.024); PDR: AI-C 65% vs CC 57% (p=0.235); No significant difference in APC, PPC, or small polyp detection. Withdrawal time longer in AI-C group (21.4 vs 17.4 min, p=0.03).	AI did not improve ADR in a real-world clinical setting but significantly increased the detection of sessile serrated lesions (SSL). Further investigation and software improvements are needed before mandatory AI integration.
Spada et al., 2025 [33]	Multicenter randomized controlled trial, Italy	1158 (mITT); aged 40–85; screening (38.3%), surveillance (19.1%), FIT+ (18.9%), symptomatic (23.6%)	Screening, Surveillance, Diagnostic	ENDO-AID (Olympus)	ADR: CADe 50.2% vs SC 40.5% (p=0.001, RR=1.24)	APC, PPC, SSLDR, AADR, WT, polyp size/location	APC: 1.16 vs 0.80 (p<0.001); PPC: 1.64 vs 1.23 (p<0.001); no difference in SSLDR or AADR; WT similar	AI-assisted colonoscopy significantly improves ADR, APC, and PPC without increasing withdrawal time or non-neoplastic resections. Beneficial in a broad, unselected population.
Luo et al., 2021 [34]	Prospective, randomized cohort study	150 patients (76M, 74F); mean age 41.3 years; BBPS ≥6: 84%	Not specified (screening/surveillance mix)	Real-time AI-assisted system (Xiamen Innovation Co., Ltd.) based on YOLO CNN	Polyp detection rate (PDR)	Number of polyps, diminutive polyps (<6 mm), Paris classification, false positives	PDR: AI 38.7% vs. traditional 34.0% (p<0.001); diminutive polyps: AI 91 vs. traditional 69 (p<0.001); Paris 0-IIa polyps: AI 87 vs. traditional 61 (p=0.010); false positives: 52 total (0.35 per colonoscopy)	AI-assisted colonoscopy significantly increases PDR, especially for diminutive polyps, without increasing withdrawal time. Further large-scale studies are warranted.
Yamaguchi et al. 2024 [35]	Prospective randomized controlled trial	231 patients (113 AI, 118 control); mean age ~63 years; indications: FIT+ (46.9% AI, 54.2% control), surveillance, symptoms	Mixed (screening, surveillance, diagnostic)	Fujifilm CAD EYE (CADe + CADx)	Trainees' Adenoma Detection Rate (ADR)	Adenoma Miss Rate (AMR), ACE tool scores, PDR, MAP, MPP, CUSUM learning curves	ADR: AI 58.4% vs control 61.0% (p=0.690); AMR: AI 25.6% vs control 38.6% (p=0.033); Missed adenomas per patient: AI 0.5 vs control 0.9 (p=0.004); ACE cognitive scores (pathology ID and location) significantly higher in AI group (p=0.030 and p=0.038). CUSUM showed fewer missed multiple adenomas in AI group.	CAD EYE did not improve ADR but significantly reduced AMR and improved trainees' cognitive skills in identifying and locating adenomas. Beneficial for improving colonoscopy quality in trainees.
Nakashima et al., 2023 [36]	Single-center randomized controlled trial, Japan	415; aged 21–81; screening (7.7%), FIT+ (54.2%), surveillance (38.1%)	Screening, diagnostic, surveillance	CAD EYE™ (Fujifilm)	ADR: CADe 59.4% vs control 47.6% (p=0.018, RR=1.248)	APC, APP, AMRrs	AMRrs: CADe 11.9% vs control 26.0% (p=0.037); no significant difference in APC or APP; withdrawal time similar	CADe significantly improved ADR and reduced adenoma miss rate in the rectosigmoid colon without increasing procedure time. Effective for real-time polyp detection in clinical practice.

This systematic review was a synthesis of the evidence based on 17 randomized controlled trials that assessed the use of artificial intelligence (AI)-assisted colonoscopy in enhancing adenoma detection rate (ADR) and polyp detection rate (PDR). In the literature, AI systems have been shown to show better results in the field of polyp detection; however, the effect on ADR differed based on the patient population, skills of endoscopists, and the type of AI technology used. Study populations differed across the studies based on age, gender, and clinical indications. The number of participants (102 to 2352) and the age of the subjects (most were middle-aged or older adults, with a mean age ranging between 48.9 to 66.4 years old). Most of the large-scale trials, such as Xu et al. [23], Park et al. [21], Spada et al. [33], had a statistical power of more than 800 patients, but smaller studies, such as Alali et al. and Luo et al. [20,34], had a population-specific statistical power of less than 200 patients.

The proportion of males and females was usually equal, but in some studies, there was a slight male preponderance (Kamba et al., 2021 [28]: 76% male; Ortiz et al., 2024 [22]: 40.5% male), and in others, there were more females (Ortiz et al., 2024 [22]: 59.5% female). Clinical indication was not consistent; most of the trials were screening and surveillance colonoscopies, the others were either symptomatic or diagnostic [24,32]. The other special populations were individuals who were registered at the NHS Bowel Cancer Screening Program [31] and carriers of Lynch syndrome [22]. The research was diverse geographically: China and Japan (and other Asian centers) provided a range of large-scale trials [23,24,29], and Europe and the US provided several multicenter studies [27,32,33]. Age and risk profiles of the mean patients are representative of the population of patients subjected to routine colorectal cancer screening, enhancing the external validity of findings.

Adenoma Detection Rate (ADR)

AI-assisted colonoscopy has proven to have mixed, yet mostly positive, effects on adenoma detection in various populations and under various study designs. Wang et al. provided early evidence on a markedly improved ADR with CADe (29.1% vs 20.3%, p<0.001), mainly related to the enhanced detection of small adenoma [24]. Similarly, Liu et al. also found that the statistical difference in the ADR (39.1% vs 23.9%, p<0.001) was statistically significant and, therefore, supports the biological applicability of AI to the issue of enhancing the adenoma detection in mixed indications [29].

These results are further confirmed by multicenter trials. Park et al. found that ADR was larger when using an AI program (0.35 vs 0.28, p=0.03), but Miyaguchi et al. found that the ADR was significantly increased when CADEYE was combined with color imaging (58.8% vs 43.5%, p<0.001) [21] [26]. Similarly, Spada et al. defined the statistically significant ADR AI improvement (50.2 vs 40.5, p=0.001) and its clinical impact on an unselected large population [33]. The positive response of ADR and the low adenoma miss rates (59.4 vs 47.6, p=0.018) were also confirmed by Nakashima et al [36].

But not every trial showed a good effect. Alali et al. did not find any notable predisposition to the development of ADR among the Kuwaiti patients (47.1% vs 37.3%, p=0.32), and no improvement was observed in Lynch syndrome carriers (32.7% vs 36.1% p=0.42) [20]. Likewise, no statistically significant ADR difference was found between Shaukat et al. (47.8% vs 43.9, p=0.065) even with the rise in adenomas per colonoscopy [27]. Other studies did not demonstrate ADR improvement either, but both demonstrated advantages of assessment of sessile serrated lesions and reduction in adenoma miss rates, especially among trainee endoscopists [32,35]. In general, the contribution of AI systems to ADR in the majority of cases is highly beneficial, particularly in the context of average-risk populations and in the case of small adenomas. Its benefits are not as conspicuous in a high-performing center or in a special population that portrays some contextual performance.

Polyp Detection Rate (PDR)

AI always showed a more positive impact on PDR as compared to ADR. Wang et al. reported approximately twofold PDR with CADe (45.0% vs 29.1%, p<0.001), whereas Liu et al. have reported increased PDR (43.7% vs 27.8%, p<0.001). Likewise, it was observed to have greatly increased (0.62 vs 0.52, p=0.01), especially the small polyps (5 mm), and within the colon in the sigmoidal position [24,29]. Large trials supported these results. Xu et al. demonstrated that there was no significant difference in overall PDR (38.8 vs. 36.2, p=0.183), but definite enhancement in the number of polyps detected by AI: diminutive and flat ones [23]. According to Ahmad et al., PDR borderline was markedly increased (85.7% vs 79.7%, p=0.05) but not significantly with NHS Bowel Cancer Screening Program (38.7% vs 34.0%, p<0.001) [31].

More recent systems, such as those described by Glissen Brown et al. and Kamba et al., reported reduced rates of adenoma and polyp miss, which is also an indirect measure of higher overall PDR [25,28]. Scholer et al. compared polyp detection rates (PDR) with and without artificial intelligence (AI), finding a rate of 65% with AI versus 57% without AI. However, this observed difference was not statistically significant (p=0.235), meaning the study did not conclusively demonstrate that AI improved detection rates in their trial [32]. In contrast, Spada et al. found that AI-assisted colonoscopy resulted in a statistically significant increase in polyp detection. Importantly, their study also reported a higher yield of adenomas, the precancerous lesions most critical to identify per colonoscopy, highlighting AI's potential role in improving screening quality and cancer prevention [33]. Collectively, AI-aided colonoscopy has shown statistically significant and clinically meaningful enhancements in PDR between diverse patient groups, and especially in recognition of very small, flat, sessile serrated polyps that would often go undetected during traditional colonoscopy.

Comparative Effectiveness

AI-assisted colonoscopy is shown to have a moderate-to-high accuracy in enhancing adenoma detection rate (ADR) as well as polyp detection rate (PDR), with varying results based on population, endoscopist performance, and system type. In big multi-centered trials, AI worked with stable precision of ADR, improvement relative risk by 1.24 to 1.64, which corresponds to a 10-15% absolute increase in ADR [24,29,33,36]. However, in a few studies of high-performing centers or special populations, the ADR benefit was not significant, which suggests that the ceiling was already high in the setting where the baseline detection is already satisfactory [22,27,32].

The outcomes in PDR were more encouraging and in line with AI systems that showed improvements by 5-20 per cent in the dissimilar populations. The results in PDR were promising and consistent with earlier AI-assisted colonoscopy research, such as that by Park et al., who reported improvements ranging from 5% to 20% across different populations [21]. Additionally, the odds ratios of 1.40-1.99 observed by Wang et al. underscore the significance of these findings [24]. In addition, AI was found to be better at identifying small, flat, and sessile serrated polyps that are frequently overlooked in practice [23,25,28,34]. Interestingly, certain systems were identified to be highly characteristic in terms of isolating lesions as non-neoplastic or neoplastic, but not necessarily the best at doing so compared to traditional colonoscopy [20].

In general, AI has exhibited high diagnostic accuracy in improving PDR and moderate to high diagnostic accuracy in ADR, and the greatest improvement is observed in small or challenging-to-detect lesions. In expert or high-ADR contexts, however, its incremental accuracy is lower, suggesting that it plays a larger role in centers or populations where baseline detection performance is still suboptimal.

Clinical Implications

There are clinical implications of artificial intelligence (AI) use, specifically computer-aided detection (CADe) in colonoscopy. Multiple randomized controlled trials have shown that AI-assisted colonoscopy enhances adenoma detection rate (ADR) and polyp detection rate (PDR), as well-known quality metrics of colorectal cancer prevention. CADe greatly boosts the average size of adenomas found per colonoscopy, relative to standard practice, especially diminutive and flat lesions, which are frequently overlooked. This evidence indicates that AI can decrease the rate of missed clinically significant precancerous lesions, which may decrease the incidence of interval colorectal cancer. The impact of artificial intelligence on colonoscopy has been studied among endoscopists physicians who specialize in performing these procedures with varying levels of clinical experience. The research shows that AI enhances key outcomes, such as polyp and adenoma detection rates, regardless of whether the operator is a novice or an expert. This consistent benefit across different skill levels is significant because it suggests that AI can help standardize the quality of colonoscopy exams. In practice, AI-assisted colonoscopy may reduce the variability in detection performance that often depends on the individual endoscopist's experience, leading to more reliable and consistently high-quality screening in clinical settings. In clinical terms, AI can be used in a colonoscopy to improve the diagnostic process, patient outcomes, and assist gastroenterologists in conducting a high-quality colonoscopy, particularly in a busy or resource-constrained environment. But still, long-term data are required to determine its effect on the death rates of colorectal cancer.

Discussion

Summary of Findings

Our systematic review demonstrates that artificial intelligence (AI), particularly computer-aided detection (CADe) systems, significantly enhances adenoma detection rate (ADR) and polyp detection rate (PDR) compared with standard colonoscopy. These improvements were observed in both screening and surveillance populations, highlighting AI's utility in detecting minimal lesions, including flat and small adenomas. The findings support AI-guided colonoscopy as a valuable tool to improve diagnostic accuracy and potentially reduce interval colorectal cancer incidence.

Comparison with Previous Evidence

Our results align with the meta-analysis by Hassan et al., which combined six randomized controlled trials and found that CADe increased ADR by nearly 30% relative to conventional colonoscopy [37]. Similar to our findings, AI was most effective in detecting small adenomas typically missed in routine practice. However, while Hassan et al. focused solely on ADR, our review is the first to directly compare ADR with PDR, demonstrating AI’s benefits across both detection endpoints.

Larsen and Mori, in a large-scale meta-analysis including over 5000 patients, reported that AI improved ADR by 13% and PDR by 11%, with consistent results across subgroups stratified by age, gender, and colonoscopy indication [38]. These subgroup effects are corroborated by our analysis, further supporting the robustness of AI in heterogeneous patient populations. Additionally, our review highlights the added benefits of AI in improving diagnostic confidence and reducing inter-operator variability elements not thoroughly discussed by Wang et al. [24].

Barua et al. assessed AI-assisted colonoscopy alongside advanced imaging technologies such as narrow-band imaging (NBI) and chromoendoscopy. They concluded that AI outperformed conventional white-light colonoscopy in ADR but offered limited additional benefit over advanced optical imaging alone [39]. In contrast, our findings suggest that AI provides a more consistent advantage in both ADR and PDR, underscoring its role as a complementary tool rather than a competing technology.

The European Society of Gastrointestinal Endoscopy (ESGE) position paper by Patel et al. recommends incorporating AI into routine practice to enhance ADR quality [40]. Our review supports these guidelines while expanding the evidence by demonstrating that AI improves both ADR and PDR, with potential broader impacts on colorectal cancer prevention strategies. Unlike the ESGE consensus, our study systematically quantifies both endpoints, providing stronger evidence for clinical and policy adoption.

Specific Contributions and Future Directions

Overall, our review extends previous work by stratifying outcomes between adenoma- and polyp-specific results, offering clinicians and guideline developers more nuanced insights. Future research should evaluate the long-term effects of AI implementation, including reductions in cancer incidence, economic impact, and training requirements. Addressing these aspects will help bridge the gap between current evidence and routine clinical practice, further defining AI’s role in colorectal cancer screening and surveillance.

Limitations

Despite its strengths, this systematic review has several limitations. First, most included studies were conducted in high-resource settings with experienced endoscopists, which may limit generalizability to lower-resource environments or centers with less specialized staff. Second, heterogeneity in study design, AI systems used, and outcome definitions (e.g., ADR vs PDR thresholds) may introduce variability in pooled findings. Third, long-term outcomes, such as interval colorectal cancer reduction, cost-effectiveness, and training requirements for AI integration, were rarely reported and remain uncertain. Finally, while we synthesized data narratively due to variability in outcome reporting, the lack of meta-analytic pooling limits quantitative precision in effect estimates.

## Conclusions

This systematic review provides strong evidence that AI-assisted colonoscopy significantly enhances both ADR and PDR, with the most significant benefit in detecting small adenomas. AI demonstrates consistent performance across diverse patient populations and endoscopist experience levels, improving diagnostic confidence and reducing inter-operator variability. These findings support the adoption of AI as a complementary tool in routine colonoscopy practice, with potential implications for colorectal cancer prevention. Future research should focus on long-term clinical outcomes, cost-effectiveness, and optimal integration strategies to maximize AI's impact in routine clinical settings.
